# Addressing Challenges in Port Depth Analysis: Integrating Machine Learning and Spatial Information for Accurate Remote Sensing of Turbid Waters

**DOI:** 10.3390/s24123802

**Published:** 2024-06-12

**Authors:** Xin Li, Zhongqiang Wu, Wei Shen

**Affiliations:** 1 School of Marine Science, Shanghai Ocean University, Shanghai 201306, China; x-li@shou.edu.cn; 2 Marine Surveying and Mapping Engineering and Technology Research Center, Shanghai 201306, China; 3 School of Information Science and Technology, Hainan Normal University, Haikou 571158, China; wuzhongqiang@hainnu.edu.cn; 4 States Key Laboratory of Satellite Ocean Environment Dynamics, Second Institute of Oceanography, State Oceanic Administration, Hangzhou 310012, China

**Keywords:** spatial information, machine learning, Random Forest (RF), water depth, Sentinel-2

## Abstract

Bathymetry estimation is essential for various applications in port management, navigation safety, marine engineering, and environmental monitoring. Satellite remote sensing data can rapidly acquire the bathymetry of the target shallow waters, and researchers have developed various models to invert the water depth from the satellite data. Geographically weighted regression (GWR) is a common method for satellite-based bathymetry estimation. However, in sediment-laden water environments, especially ports, the suspended materials significantly affect the performance of GWR for depth inversion. This study proposes a novel approach that integrates GWR with Random Forest (RF) techniques, using longitude, latitude, and multispectral remote sensing reflectance as input variables. This approach effectively addresses the challenge of estimating bathymetry in turbid waters by considering the strong correlation between water depth and geographical location. The proposed method not only overcomes the limitations of turbid waters but also improves the accuracy of depth inversion results in such complex aquatic settings. This breakthrough in modeling has significant implications for turbid waters, enhancing port management, navigational safety, and environmental monitoring in sediment-laden maritime zones.

## 1. Introduction

Water depth is an essential factor for port management, navigation safety, marine engineering, and environmental monitoring [1,2]. However, traditional methods of water depth measurement, such as shipborne sonar and airborne laser sounding, are often costly, time consuming, and limited by accessibility and security issues. Remote sensing technology offers an alternative and complementary way to estimate water depth from multispectral or hyperspectral images, which can cover large areas with high spatial resolution and frequent temporal revisit [3,4,5]. However, remote sensing of water depth in port areas faces some challenges, such as complex water quality, heterogeneous bottom types, and the influence of human activities [6,7,8].

Various methods have been proposed to invert water depth from remote sensing images, which can be broadly classified into two categories: empirical methods and analytical methods [9]. Empirical methods are based on the statistical relationship between water depth and image reflectance, which are usually simple and fast but require sufficient in situ measurements for calibration and validation [10,11]. Analytical methods are based on the physical model of radiative transfer in water, which can account for the effects of water quality and bottom reflectance but require the knowledge of many optical parameters that are difficult to obtain or estimate [4,12,13,14,15]. Among the empirical methods, the log-linear method [10,16,17] and the log-ratio method [18] are the most widely used, which assume a linear or logarithmic relationship between water depth and image reflectance in two bands. However, these methods may not be suitable for port areas with turbid water and diverse bottom types, which may introduce nonlinearities and uncertainties in the inversion process.

Machine learning algorithms have been increasingly applied to remote sensing of water depth, as they can capture the complex and nonlinear relationship between water depth and image reflectance without relying on physical models or optical parameters [19,20,21,22,23]. Among them, Random Forest (RF) is a popular and powerful algorithm that can handle high-dimensional and noisy data with high accuracy and robustness. RF is an ensemble learning method that combines multiple decision trees to make predictions based on a majority vote or an average value. RF has been successfully applied to water depth inversion in various environments, such as coral reefs, lakes, and coastal zones. However, RF does not consider the spatial information of the image pixels, which is related to the depth of the water closely.

However, most of the existing methods for water depth inversion assume that the reflectance of water is spatially homogeneous and independent of the location [24,25]. This assumption may not be valid in some cases, especially in coastal areas where the water quality and bottom characteristics may vary significantly across space. Therefore, it is necessary to consider the spatial heterogeneity and autocorrelation of water reflectance in water depth inversion [26]. One possible way to do this is to use geographically weighted models (GWMs), which are spatially varying regression models that can account for the local variations of the relationships between variables [27]. GWMs have two main advantages: one is that they can capture the spatial non-stationarity of the data, i.e., the data at different locations may have different statistical characteristics; the other is that they can provide local parameter estimates for each location, thus reflecting the spatial details and differences [28]. GWMs have been applied to various fields of remote sensing, such as land cover classification, vegetation mapping, urban sprawl analysis, and soil moisture estimation.

In areas of turbid waters, especially around ports, the effectiveness of depth inversion models that rely on geographically weighted methods can be greatly diminished by factors such as suspended sediments. However, there exists a pronounced relationship between water depths and their geographical coordinates in these aquatic environments. Addressing the interference caused by materials suspended in the water to accurately determine depth values and improve the accuracy of depth inversion is a critical challenge in the field of port depth analysis.

In this paper, we propose a novel method for water depth inversion in port areas based on RF and geographically weighted regression (GWR). After GWR is performed, we bring the latitude and longitude data into the Random Forest model as characteristic factors and compare it with the traditional model. By incorporating GWR into RF, we can account for the spatial heterogeneity and autocorrelation of water depth and image reflectance in port areas. We use Sentinel-2 multispectral images as the input data and compare our method with the classical Stumpf algorithm, the log-linear algorithm, and the standard RF algorithm. We use the MATLAB toolbox to implement GWR and use the latitude and longitude coordinates as additional features for RF. We test our method at Sanya Nanshan Port in Hainan Province, China, which is a typical port area with complex water quality and bottom types. The results show that our method achieves higher accuracy than the other methods and can effectively deal with the complex water quality and bottom types in port areas.

The rest of this paper is organized as follows. Section 1 introduces the theoretical background and principles of GWM and RF. Section 2 describes the data sources and preprocessing steps. Section 3 presents the methodology and implementation details of our method and the comparison methods. Section 4 reports the results, and Section 5 reports a discussion of our case study. Section 6 concludes this paper and suggests some future work.

## 2. Materials

### 2.1. The Study Areas and Satellite Imagery

In our extensive survey, we investigated the unique turbidity at Nanshan Port, situated in Hainan Province, China. Researchers aim to understand its impact on ecosystems, fisheries, and port operations. The phenomenon of turbid waters involves intricate processes, making Nanshan Port a crucial site for scientific inquiry [6,29].

**Figure 1:**
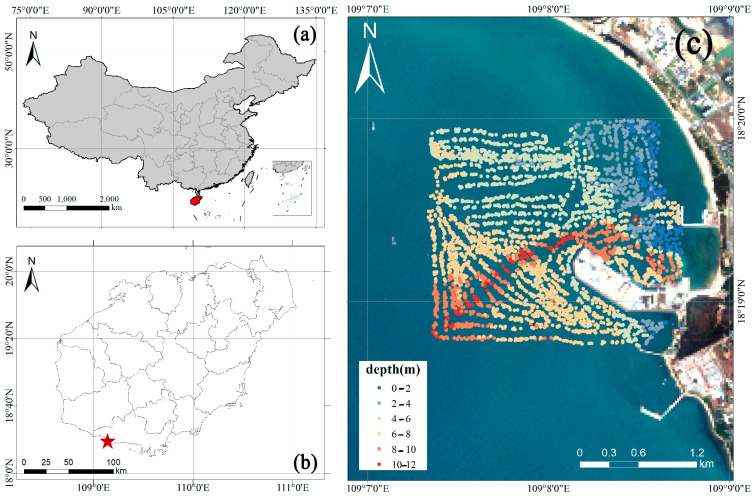
The location of the port and bathymetric rendering by sonar. (**a**) The location of Hainan Island, (**b**) the location of Nanshan Port, (**c**) the location of Nanshan Port Remote sensing data and measured data. (The red part of (**a**) is Hainan Island, and red star in (**b**) represents the location of Nanshan Port).

Furthermore, as Figure 1 shows, Nanshan Port’s significance extends beyond its research potential, as it serves as a critical port and logistical hub. Its strategic geographical location positions it as a key nexus for maritime transportation and trade. Scientists and researchers are also studying the port’s operational aspects, water management strategies, and sustainable development to ensure its continued role in the region’s economy and ecological systems. Nanshan Port’s distinctive characteristics, marked by its turbid waters and its dual role as a research hub and a vital port, have placed it in the spotlight. This convergence of factors presents invaluable opportunities for scientific exploration and development while exerting profound and far-reaching effects on the region’s economy and ecosystems [6,29].

We used satellite data from the Sentinel-2 collected on 17 July 2021, and we estimated bathymetry in shallow waters (up to 30 m below sea level) at Nanshan Port. The ACOLITE software, version Python 20190326.0, provided remote sensing reflectance (Rrs) data for all visible and near-infrared bands at a 10-m spatial resolution [17,18]. We employed multiple spectral reflectance bands from Sentinel-2 to create a bathymetry map [19]. Additionally, we also removed sun glint effects with the same software [20,21].

### 2.2. In Situ Data

Acoustic measurements were conducted at Nanshan Port from July 11 to 13, 2021. The equipment used included the R2Sonic 2024 broadband multibeam echo sounder, Octans integrated gyroscope and motion sensor, Trimble Beacon differential global positioning system (GPS), and Teledyne Odom surface sound speed probe and sound speed profiler. Notably, the differential GPS module facilitated navigation and positioning in multibeam bathymetry and marine scanning, with a nominal dynamic accuracy of ±1 m. The R2Sonic 2024 detector, a 5G broadband high-resolution shallow water multibeam system, provided detailed data. Additionally, the Octans, equipped with fiber optic compass and motion sensors, ensured precise measurements. Wind speeds ranged from 2 to 3 levels and wave heights were between 0.1 and 0.2 m, meeting the requirements for satellite bathymetry. Tidal offset correction relied on data from the China Maritime website [6,29].

## 3. Methods

### 3.1. Water Depth Retrieval Model

#### 3.1.1. Stumpf Model

Stumpf et al. [18] proposed a model based on the log conversion ratio as follows:

(1) $$Z=m_{1}\frac{\ln\left[nR\left(\lambda_{i}\right)\right]}{\ln\left[nR\left(\lambda_{j}\right)\right]}+m_{0}$$

where *n* is 1000; *m*_0_ and *m*_1_ are the regression coefficients; and *R*(*λ_i_*) and *R*(*λ_j_*) are the remote sensing reflectance of the blue band *i* and the green band *j*.

#### 3.1.2. Log-Linear Model

The log-linear model [10,16,17] formula is as follows:

(2) $$Z=a_{1}\ln\left[L\left(\lambda_{i}\right)-L_{\infty }\left(\lambda_{i}\right)\right]+a_{2}\ln\left[L\left(\lambda_{j}\right)-L_{\infty }\left(\lambda_{j}\right)\right]+a_{3}$$

*L*(*λ_i_*) and *L*(*λ_j_*) are the radiance of band *i* and band *j*; *a*_1_, *a*_2_, and *a*_3_, are the regression coefficients; and *L*∞(*λ_i_*) and *L*∞(*λ_j_*) are the radiance of each band in deep water.

#### 3.1.3. Random Forests (RFs)

Random Forest (RF) is a robust ensemble learning algorithm that combines decision trees through bagging and random subspace methods [30,31]. It is adept at handling high-dimensional, noisy data for classification and regression tasks, providing variable importance measures for feature contribution [32].

RF has been successfully applied to water depth inversion in various environments, such as coral reefs, lakes, and coastal zones. RF can capture the complex and nonlinear relationship between water depth and image reflectance without relying on physical models or optical parameters. RF can also handle missing values and outliers and avoid overfitting and multicollinearity problems.

The RF model setup involves several key parameters:

- Number of Trees: 100;
- Maximum Depth of Trees: 20;
- Minimum Samples per Leaf: 1;
- Bootstrap Sampling: Enabled.

These parameters were selected based on preliminary experiments to balance model complexity and computational efficiency.

### 3.2. Geographically Weighted Models (GWM)

Geographically weighted regression (GWR) is a spatial analysis technique that extends traditional linear regression methods by explicitly considering spatial heterogeneity. Unlike conventional linear regression models, which assume that the relationships between variables are constant across space, GWR recognizes the presence of spatially varying relationships [24,26,27,28].

GWR accomplishes this by assigning spatially varying weights to neighboring data points based on their proximity to a target location. Closer observations are given greater weight, while those farther away contribute less to the regression model. The outcome is a set of locally calibrated regression models, each tailored to a specific geographic area. This spatially adaptive approach enables a more precise characterization of the varying relationships between variables across the study area [24,26,27,28].

At its core, GWR is based on the premise that local conditions and interactions can significantly influence the relationships between variables. Therefore, it is particularly well-suited for applications where spatial variability and context play a crucial role, such as water depth remote sensing inversion [24,26,27,28].

### 3.3. GWR-RF-Lat./Lon (GWR-RF2)

In turbid aquatic environments, particularly harbors, factors like suspended sediment can undermine the performance of depth inversion models grounded in geographically weighted approaches. Despite these challenges, a notable correlation exists between the depths of waters and their geographical positions in these maritime settings. Addressing the obstacles presented by suspended materials to accurately ascertain depth measurements and refine the fidelity of depth inversion stands as a pivotal concern in the advancement of harbor depth assessment techniques.

In this paper, we propose a novel method for water depth inversion in port areas based on RF and geographically weighted regression (GWR). Geographically weighted regression (GWR) is a spatial analysis technique that extends traditional regression models by considering spatial heterogeneity. This means that instead of assuming a constant relationship between variables across space, GWR allows the regression coefficients to vary locally based on the geographical location of each observation. This approach captures the spatial non-stationarity in the data, making it particularly useful for applications like water depth inversion in turbid waters, where local conditions can significantly impact the relationships between variables.

Random Forest (RF) is an ensemble learning method known for its ability to handle complex and nonlinear relationships between input features and the target variable. It operates by constructing multiple decision trees and aggregating their results, which enhances the model’s robustness and accuracy, particularly in high-dimensional and noisy datasets.

#### 3.3.1. Integration of Latitude and Longitude

The GWR-RF-Lat./Lon method integrates GWR and RF by incorporating latitude and longitude as additional features in the RF model. The traditional GWR-RF approach utilizes GWR to account for spatial variability in the relationships between variables, followed by RF to model the complex interactions between input features and water depth. However, this traditional method might not fully exploit the spatial information inherent in geographical coordinates.

By explicitly including latitude and longitude as input features in the RF model, the GWR-RF-Lat./Lon method enhances the model’s ability to leverage spatial information. This inclusion allows the RF model to directly learn from the geographic context of each data point, thereby improving the accuracy of depth inversion in turbid waters. This approach addresses the challenges posed by suspended sediments and other local environmental factors that can affect the performance of depth inversion models.

#### 3.3.2. Methodology

1. Data Preprocessing: The input data, including multispectral remote sensing reflectance from Sentinel-2 and in situ depth measurements, are preprocessed. Atmospheric corrections are applied to ensure accurate reflectance values.
2. GWR Implementation: GWR is applied to the input data to account for spatial heterogeneity in the relationship between remote sensing reflectance and water depth. The GWR model produces locally calibrated regression coefficients for each geographic location.
3. Feature Engineering: Latitude and longitude coordinates are added as input features to the RF model. This step allows the RF model to consider the spatial context of each observation directly.
4. RF Model Training: The RF model is trained using the combined features, including remote sensing reflectance, latitude, and longitude. The model learns the complex and nonlinear relationships between these features and water depth.
5. Depth Inversion: The trained GWR-RF-Lat./Lon model is used to predict water depths across the study area. The predictions are evaluated against in situ measurements to assess the model’s accuracy.

#### 3.3.3. Advantages over the Traditional GWR-RF Method

Enhanced Spatial Awareness: By incorporating latitude and longitude directly into the RF model, the GWR-RF-Lat./Lon method enhances the model’s ability to leverage spatial information, resulting in more accurate depth predictions in spatially heterogeneous environments.

Improved Accuracy: The inclusion of geographic coordinates helps mitigate the impact of suspended sediments and other local factors that can distort depth inversion results, particularly in turbid waters.

Flexibility: This method can be adapted to various remote sensing applications beyond water depth estimation, such as water quality assessment and atmospheric studies, by including relevant geographic features.

### 3.4. Accuracy Evaluation Methods

The evaluation metrics for water depth accuracy are R-squared (R^2^) and the root-mean-square error (RMSE), with respective formulas provided [32] as follows:

(3) $$RMSE=\sqrt{\frac{\sum_{i=1}^{n}{\left(Z_{i}-Z_{i}^{\prime }\right)}^{2}}{n}}$$

$Z_{i}$ is the estimated water depth; $Z_{i}^{\prime }$ is the actual water depth; and n is the number of water depth points.

### 3.5. Bathymetry Mapping

**Figure 2:**
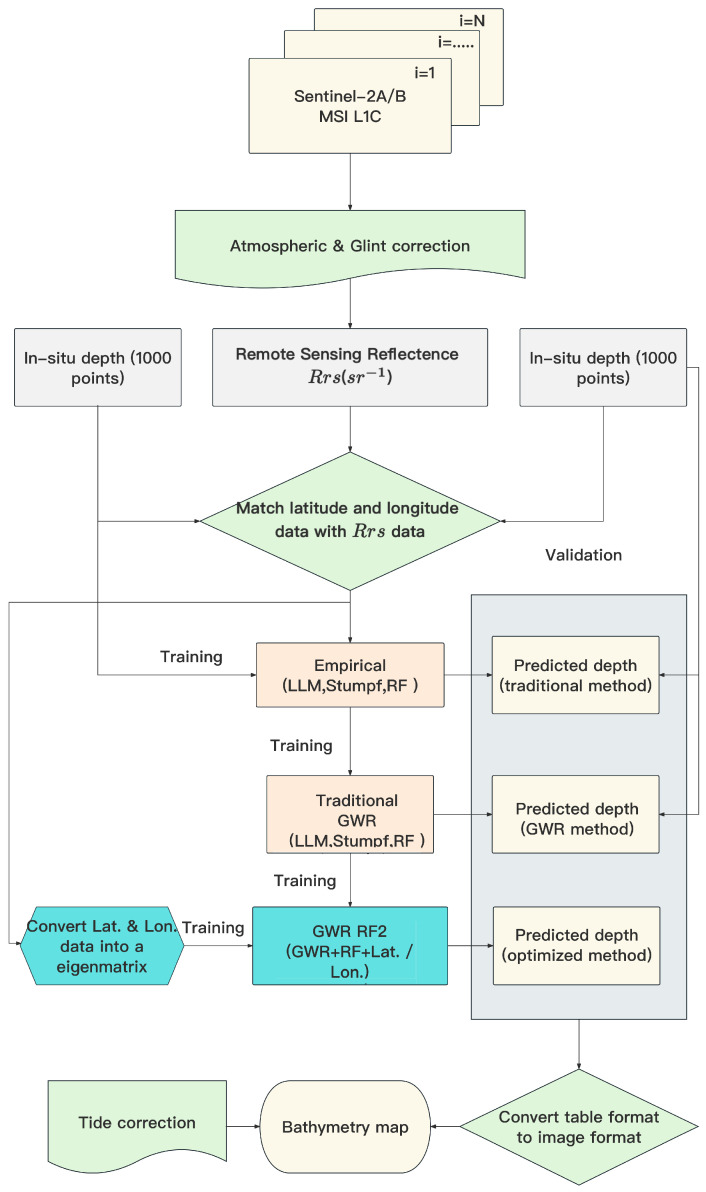
The workflow of the bathymetry method.

In this study, as Figure 2 shows, the approach involves two stages. First, we select high-quality remote sensing images and apply atmospheric correction to ensure accurate reflectance data. We then estimate water depth using various bathymetry algorithms, considering tide effects for consistency. In the second stage, we convert and utilize data for training and prediction, including processing Sentinel-2 data into a tabular format and matching the latitude and longitude with *R*_rs_ data. Then, we predict the depth with the GWR model and the RF model. The performance assessment involves comparing predicted depths with in situ measurements, demonstrating significant error reduction and enhanced accuracy. By structuring our approach into these stages and optimizing data usage, we developed a reliable method with potential applications requiring precise water depth information.

The necessity of tidal correction arises from the fact that our in situ data were collected over several days under varying tidal conditions. The remote sensing data were acquired on 17 July 2021, while the in situ measurements were conducted from July 11 to 13, 2021. Tidal variations can significantly impact water depth readings, making it essential to correct the in situ data to a common reference level. This correction ensures temporal consistency between the in situ measurements and the remote sensing data, providing a reliable basis for comparison.

## 4. Results

In this study, we employed three models, Stumpf, log-linear, and RF (Random Forest), which were trained with remote sensing reflectance from varied spectral bands. To ensure dataset diversity, we used spatial random sampling. Our research focused on water depth estimation accuracy using a geographically weighted model (GWM) on a Sentinel-2-derived dataset of 4000 points. We used outputs from the Stumpf, log-linear, and RF models for geographically weighted regression, training 4000 and validating 1000 points, compared to actual values. Additionally, we introduced a novel geographically weighted model incorporating latitude and longitude as features, gathering corresponding remote sensing reflectance data across different spectral bands. Key metrics, like RMSE and R^2^, were computed. Rigorous quality checks were conducted. Potential extensions included depth mapping in specific coastal regions. This approach offers insights into machine learning algorithms and Sentinel-2 imagery for water depth estimation, with implications for future accuracy improvements. All experiments were conducted in MATLAB, with sample points extracted randomly for training and validation. Calibration samples were selected randomly based on depth distribution. RMSE and R^2^ values were averaged across samples for the final presentation.

**Figure 3:**
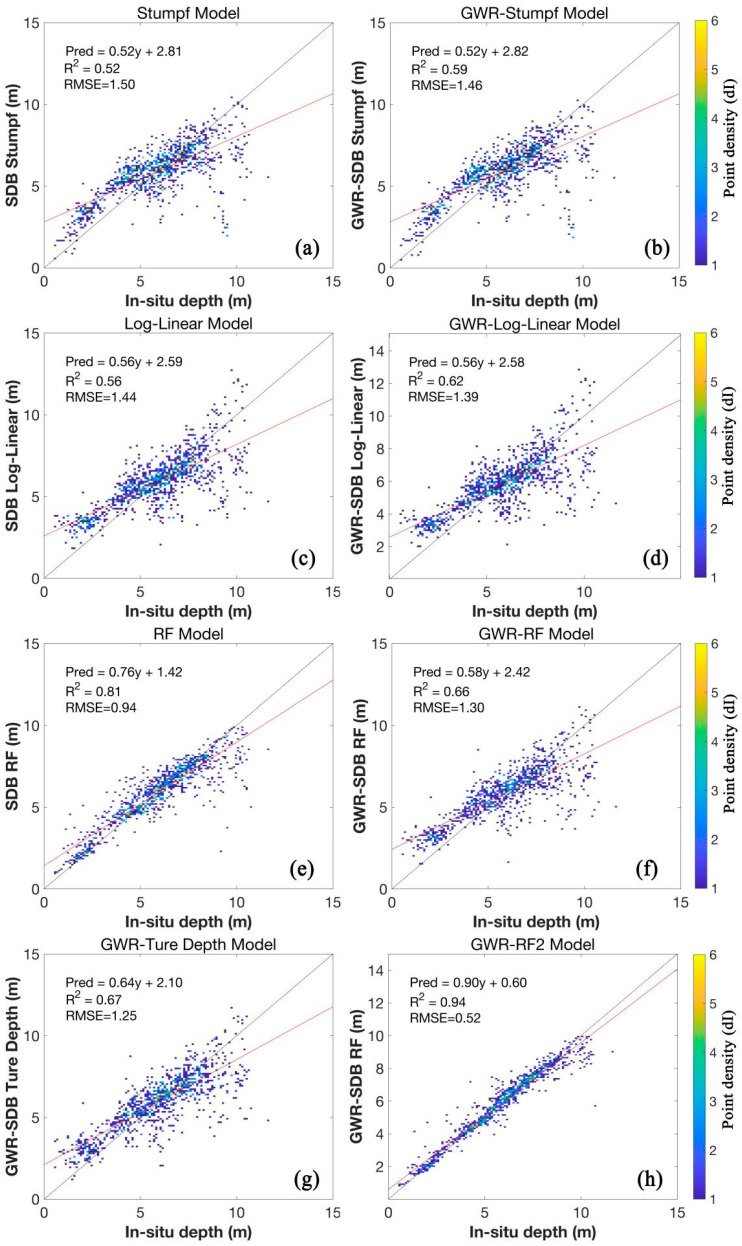
Bathymetry result between the in situ depths and predicted data. (**a**) Stumpf; (**b**) GWR-Stumpf; (**c**) Log-liner; (**d**) GWR-Log-liner; (**e**) RF; (**f**) GWR-RF; (**g**) GWR-Ture Depth Model; (**h**) GWR-RF-Lon./Lat. Grey stands for the 1:1 line, pink stands for the regression line of the scatter plot.

As Figure 3 shows, the notable variations in water depth estimates across algorithms. The geographically weighted model performed best, followed by log-linear, with Stumpf being less accurate. Incorporating geographic features, like latitude and longitude, enhanced model accuracy significantly.

The geographically weighted Random Forest (GWR) model, integrating latitude and longitude as features, demonstrates high accuracy in water depth retrieval, with data points closely aligned to the 1:1 line with minimal scatter. In contrast, the Stumpf model exhibits the lowest accuracy, while the log-linear model falls in between.

Utilizing the Random Forest model’s results as input for the GWR model yields lower accuracy than the original outcomes, possibly due to variations in remote sensing reflectance in turbid waters. Notably, the GWR model excels in the 10-m depth range, exhibiting low RMSE and high R^2^ values.

**Figure 4:**
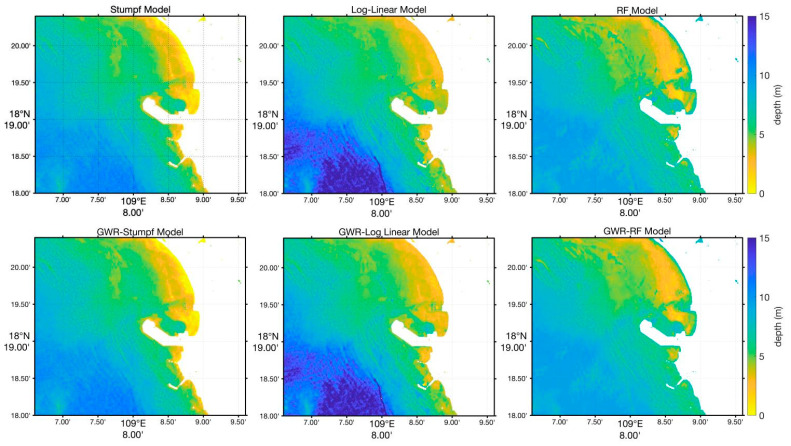
The bathymetry map estimated based on different bathymetry methods.

The results depicted in Figure 4 illustrate differences in accuracy, particularly in very shallow (<0.5 m) and very deep (>15 m) waters near the coastline. The Random Forest model closely mirrors actual conditions across shallow to deep waters, showcasing superior accuracy in water depth estimation compared to the other models.

While all models capture the general trend of water depth variations, minor differences emerge in finer details. For instance, the Stumpf model effectively addresses seabed geological heterogeneity impacts, unlike the log-linear algorithm. However, in turbid harbor areas, the log-linear model outperforms.

In the 3–8 m depth range, all models exhibit consistent trends, yet the Stumpf model and the log-linear algorithm show more noisy data points within a triangular region compared to the Random Forest model. In water depths exceeding 10 m, especially in harbor navigation channels, the GWR model with latitude and longitude features yields more accurate results.

This approach overcomes the limitations of geographically weighted models in turbid waters, significantly enhancing depth inversion accuracy, particularly in such challenging environments. By considering geographic factors, the proposed method provides more reliable depth inversion results, crucial for applications in turbid waters and port areas.

The experimental results indicate that traditional models, such as the Stumpf and log-linear models, perform poorly in turbid water conditions. This is primarily due to their high sensitivity to water turbidity. In environments with high levels of suspended sediments, these models struggle to accurately invert water depth because the suspended particles significantly scatter and absorb light, leading to inaccuracies. Additionally, these traditional models assume a linear or logarithmic relationship between spectral reflectance and water depth, which is often complex and nonlinear in turbid waters. Moreover, the Stumpf and log-linear models do not account for the spatial heterogeneity and autocorrelation of water depth and reflectance, which is particularly problematic in port areas where water quality and bottom characteristics vary significantly over short distances.

**Figure 5:**
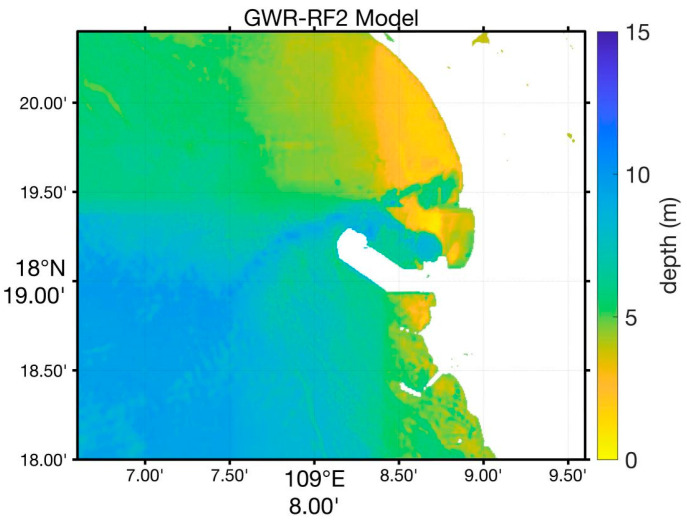
The water depth map estimated based on the GWR-RF-Lat./Lon method.

In contrast, the proposed GWR-RF-Lat./Lon model, as Figure 5 shows, which incorporates latitude and longitude as features, significantly improves inversion accuracy in turbid waters. By including geographic coordinates as input features, this model effectively captures the spatial variations in water depth, providing more accurate and reliable depth estimates, even in the presence of suspended sediments. The combination of Random Forest and geographically weighted regression allows the model to capture the complex and nonlinear relationships between reflectance and depth. The experimental results demonstrate that our model achieves higher accuracy compared to traditional methods, as evidenced by lower RMSE values and higher R^2^ values across various depth ranges. This improvement is particularly notable in challenging turbid water conditions, where traditional models are less effective.

## 5. Discussion

### 5.1. The Performance of the Bathymetry Retrieve Model

Table 1 shows RMSE values for different depth ranges, highlighting our method’s superior overall accuracy, notably in the 6–9 m range. Despite transparency favoring optical methods in our coastal study area, all methods show increased error beyond 9 m of depth. Our method notably enhances depth estimation across the 0–12 m range, especially at 4 m of depth. A comparison with a geographically weighted Random Forest algorithm using latitude and longitude features showed no significant improvement, likely due to variations in remote sensing reflectance caused by water turbidity.

### 5.2. Bathymetry Modeling: Uncertainty and Implications

Based on the results, our proposed method achieved the best water depth inversion within the 12 m range in the study area, with low RMSE and high R^2^ values. However, we also acknowledge some uncertainties and challenges in applying remote sensing inversion algorithms for water depth estimation in turbid waters.

**Figure 6:**
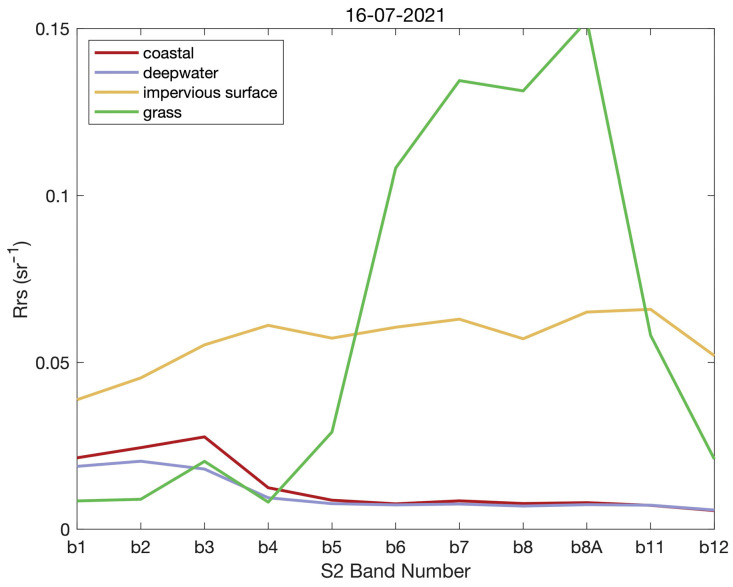
Histogram comparison of residual errors across different methods.

(a) Data Quality: For instance, noise or interference from atmospheric or water turbidity factors may introduce errors in remote sensing data. We applied ACOLITE atmospheric correction to reduce these effects and plotted the average surface reflectance values of different regions around the study area in Figure 6. The spectral shapes of each habitat (coastal, deep water, impervious surface, and grassland) show their signal differences. The spectral curves indicate that ACOLITE atmospheric correction preserved the spectral characteristics of different types of ground objects.

(b) Model Selection Impact on Inversion Precision: Although machine learning models are typically more robust compared to traditional semi-empirical, bio-optical, and semi-analytical models, employing geographically weighted regression with machine learning algorithms did not result in enhanced outcomes compared to previous approaches. This discrepancy may stem from uncertain factors, like chlorophyll and suspended matter in turbid waters.

(c) Water Environment.

**Figure 7:**
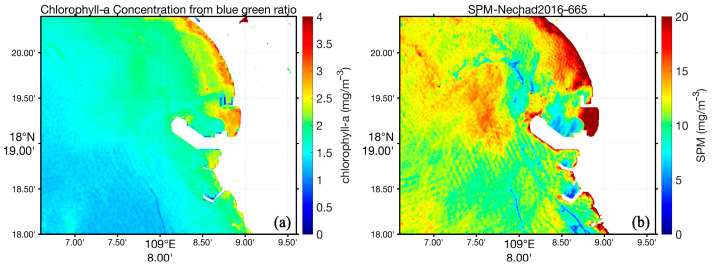
(**a**)The chlorophyll a and (**b**) suspended particulate matter (SPM) concentrations derived by ACOLITE software.

Figure 7 displays the chlorophyll a and suspended particulate matter (SPM) concentrations derived by ACOLITE software. The results reveal that the chlorophyll concentration in water ranged from 0.5 to 4 mg/m^3^. For depths exceeding 10 m, it was 1.5 mg/m^3^, and there was a correlation between the chlorophyll a concentration and water depth obtained by the blue-green ratio algorithm.

### 5.3. Complexity Analysis of the Model

To evaluate the complexity of the proposed model, we considered factors such as training time and the quantity of parameters. The model was implemented using MATLAB and processed on an Apple M1 processor with 16 GB of memory. The training time for the model is approximately 20 min under these conditions. It should be noted that the processing time may vary depending on the specific dataset and the computer configuration used. For those interested in the implementation details, the MATLAB code is available upon request. However, due to the confidentiality of the port data, the raw data cannot be shared.

## 6. Conclusions

In conclusion, this paper has introduced a novel approach for water depth estimation in port areas using a combination of geographic factors with Random Forest (RF) and geographically weighted regression (GWR). The superiority of our approach lies in its ability to capture the complex and nonlinear relationships between water depth and image reflectance without relying on physical models or optical parameters. The method proposed in this paper overcomes the issue where geographically weighted models are rendered ineffective for depth inversion in turbid waters due to the impact of suspended sediments. By taking geographic factors into account, the proposed approach significantly enhances the accuracy of depth inversion results, particularly in turbid waters and port areas.

Looking ahead, the potential applications of this methodology extend beyond water depth estimation in port areas. This algorithm can be adapted and applied to various remote sensing fields, including water quality assessment and atmospheric studies. The flexibility of our approach makes it a valuable asset in addressing a wide range of research questions and practical challenges.

As we move forward, further research and development in data quality improvement, model optimization, and input variable selection will be essential to advance the accuracy and reliability of water depth estimation. Additionally, the adaptability of our approach opens new possibilities for remote sensing applications and promising innovations in fields such as water quality monitoring, atmospheric analysis, and beyond. The future is bright for the utilization of this algorithm in diverse remote sensing domains, offering solutions to complex problems and contributing to a deeper understanding of our natural environment.

## Figures and Tables

**Table 1:** A table of RMSE errors across varying water depths and different bathymetric methods.

Training Method	RMSE				
	0–3 m (127 Points)	3–6 m (367 Points)	6–9 m (200 Points)	>9 m (73 Points)	Overall (1006 Points)
Stumpf	1.26	1.17	1.08	3.73	1.50
Log-Linear	1.49	1.04	1.20	3.21	1.44
RF	0.74	0.76	0.70	2.27	0.94
Stumpf with GWR	1.25	1.18	1.08	3.74	1.49
Log-Linear with GWR	1.50	1.03	1.20	3.21	1.39
RF with GWR	1.29	0.92	1.06	3.01	1.30
Ground Truth with GWR	1.23	0.98	1.05	2.72	1.25
GWR-RF-Lat./Lon	0.49	0.46	0.35	1.19	0.52

## Data Availability

Due to confidentiality agreements, we cannot share the raw data or complete dataset used in this study. We emphasize our commitment to detailed methodology and results transparency, despite data confidentiality. Interested researchers may contact us to explore data sharing options within legal and ethical bounds.
